# Interaction Rating Scale (IRS) as an Evidence-Based Practical Index of Children’s Social Skills and Parenting

**DOI:** 10.2188/jea.JE20090171

**Published:** 2010-03-05

**Authors:** Tokie Anme, Ryoji Shinohara, Yuka Sugisawa, Lian Tong, Emiko Tanaka, Taeko Watanabe, Yoko Onda, Yuri Kawashima, Maki Hirano, Etsuko Tomisaki, Yukiko Mochizuki, Kentaro Morita, Amarsanaa Gan-Yadam, Yuko Yato, Noriko Yamakawa, Japan Children’s Study Group

**Affiliations:** 1 Research Institute of Science and Technology for Society, Japan Science and Technology Agency, Tokyo, Japan; 2 Graduate School of Comprehensive Human Sciences, University of Tsukuba, Tsukuba, Ibaraki, Japan; 3 College of Letters, Ritsumeikan University, Kyoto, Japan; 4 Clinical Research Institute, Mie-Chuo Medical Center National Hospital Organization, Tsu, Japan

**Keywords:** cohort study, social development, interaction, parenting, scale

## Abstract

**Background:**

The purpose of this paper is to describe the features of the Interaction Rating Scale (IRS) as an evidence-based practical index of children’s social skills and parenting.

**Methods:**

The participants in our study, which was conducted as part of a Japan Science and Technology Agency (JST) project, were 370 dyads of children (aged 18, 30, and 42 month) and 81 dyads of 7-year-old children with their caregivers. The participants completed the five minute interaction session and were observed using the IRS.

**Results:**

The results indicated that the IRS can measure children’s social skill development and parenting with high validity. Along with the discriminate validity for pervasive development disorder (PDD), attention-deficit/hyperactivity disorder (ADHD), abuse and maltreatment, a high correlation with the SDQ (Strength and Difficulties Questionnaire), and high reliability, the IRS is effective in describing features of social skill development.

**Conclusions:**

The IRS provides further evidence of the fact that in order to study children’s social skill development, it is important to evaluate various features of the caregiver-child interaction as a predictor of social skills.

## INTRODUCTION

The study of children’s social development has attracted caregivers, practitioners, and researchers from all over the world. Social competence is defined as the ability to understand others in the context of social interaction and to engage in smooth communication with them. Increasing numbers of impulsive behavior and maladjustment to society in school-aged children and adolescents requires society to prepare appropriate education and environments for those children.^1^ Children’s social development is determined by the complex interaction of the child themselves, their home environment, peer relationships, and the larger sociocultural environment.^2^ Accordingly, children’s social skills should be evaluated with the interaction between the child and social environment.^3^ However, the methodology that considers children in conjunction with their social environment across developmental stages has not yet been well developed.

Many researchers are focused on measuring a quality of children’s rearing environment and parenting, based on the theory that early rearing environment is significantly related to child development. Two instruments, namely, the Home Observation for Measurement of the Environment (HOME)^4^ and the Index of Child Care Environment (ICCE)^5^ are often used in research related to child development.

The HOME and the ICCE evaluate the children’s rearing environment within natural settings, which reflects the caregivers’ emotional and verbal responsiveness to the child, and the caregivers’ acceptance of the child’s behavior. The HOME has been adopted by studies conducted at the National Institute of Child Health and Human Development (NICHD) in the United States,^6^ and is also widely used in more than one hundred countries. The ICCE has been used to investigate the effect of child care on children’s development in Japan.^7^^–^^9^ In addition, the Mediated Learning Experience Rating Scale (MLERS) has been used to assess the sensitivity and teaching of adults (caregivers and teachers) toward children through observation of the adult-child interaction.^10^

The tool that is currently used to assess social competence is the Social Skills Rating System (SSRS),^11^ which was used in the study conducted at the NICHD. The SSRS evaluates children’s social competence on the basis of information provided by parents and teachers; however, this method of evaluating social competence suffers from the inevitable drawbacks of the possibility of parents and teachers missing out on or distorting information. The Nursing Child Assessment Satellite Training (NCAST), which emphasizes the role of the caregiver in the development of social competence, was developed in the United States. The validity of NCAST had been confirmed for evaluating the communication and interactional patterns between caregiver and child.^12^ It is useful to evaluate the quality of child-rearing objectively, but it was much concentrated on caregiver’s teaching skills, so cannot be used directly for assessing children’s social skills development.

The Interaction Rating Scale (IRS) can evaluate the child-caregiver interactions in a short period of time in daily situations. The inter-observer reliability of the IRS was found to be 90%.^13^

The purpose of this paper is to describe the features of Interaction Rating Scale (IRS) as an evidence-based practical index of children’s social skills and parenting.

## METHODS

### Participants

The participants of the study were 231 (aged 18 months), 344 (aged 30 months), 175 (aged 42 months) and 82 (aged 7 years) dyads of children and their caregivers, who participated in the Japan Science and Technology Agency (JST) project.

In order to comply with the ethical standards laid down by the JST, before conducting the research, the families of all the participants signed informed consent forms and were made aware that they had the right to withdraw from the experiment at anytime. As the infants were too young to provide informed consent, we carefully explained the purpose, content, and methods of the study to the caregivers and obtained their consent. To maintain confidentiality of the personal information of the participants, their personal information was collected anonymously, and a personal ID system was used to protect personal information. Further, all the image data were stored on a disk, which was password protected; only the researchers who were granted permission from the chairman were given access to the data.

This study was approved by the ethics committee of the JST.

### Measures

The IRS is used to measure the child’s social competence and the caregiver’s child rearing competence through five minute observations of caregiver-child interactions. It is appropriate for the assessment of interactions between caregivers and children from infant to eight-year-old. It includes 70 items for a behavioral score and 11 items for an impression score, grouped into ten subscales. Five subscales focus on children’s social competences: 1) Autonomy, 2) Responsiveness, 3) Empathy, 4) Motor regulation, and 5) Emotional regulation. Another five items assess the caregiver’s parenting skills: 6) Respect for autonomy development, 7) Respect for responsiveness development, 8) Respect for empathy development, 9) Respect for cognitive development, and 10) Respect for social-emotional development. And one item assesses an overall impression of synchronous relationships.

The total of 81 items was composed from several sources: original items by the study authors, several overlapping items from the HOME (Home Observation for Measurement of the Environment),^4^ the SSRS (Social Skills Rating Systems),^11^ and the NCAST (Nursing Child Assessment Satellite Training)^12^ teaching scales (36 items).

A training manual for the IRS has been developed for practitioners and researchers.^14^

Two different sets of variable are scored: behavior items and impression items for each subscale. Each subscale assesses the presence of behavior (1 = Yes, 0 = No), and the sum of all items in the subscale provides the overall behavior score.

Scores on the impression items and the overall impression item are on a five-point scale, where 1 = not evident at all, 2 = not evident, 3 = neutral, 4 = evident, 5 = evident at high level.

The evaluator completes the checklist composed of 25 items focusing on children’s behavior toward caregivers (eg, Child looks at caregiver’s face as social referencing) and 45 items focusing on the caregiver behavior. The observer then provides an impression on a 5-point scale of the level of development for each subscale and for an overall impression.

Internal consistency in each categories, as measured by Cronbach’s alpha, ranged from .43 to .88, and the total internal consistency was excellent (.85–.91).

### Procedure

In this study, the IRS was evaluated as follows: a five minute video recording of the setting of the child-caregiver interaction (the child and caregiver playing with blocks and putting them in a box) was conducted. The caregiver-child interactions were videotaped in a controlled laboratory environment. The recording was carried out in a room with five video cameras; one camera was placed at each of the four corners and one was placed in the central ceiling position. The dyads of children were escorted into a room (with dimensions of 4 × 4 meters) furnished with a small table and a small-sized chair meant for a child. The caregiver introduced herself to the child and interacted with the child in a natural manner, just as she would on a regular day.

To score the behavior, two members of the research teamed coded the behaviors observed. A third child professional, who had no contact with the participants, also scored the behavior. The behavior of the children and caregiver during the caregiver-child interaction was coded as follows. If the child displayed the behavior described in the item, a score of 1 was given; conversely, if the child failed to display the behavior described in the item, a score of 0 was given. A child’s total score was the sum of the score that he/she received on all the subscales. A higher score indicated a higher level of development. The same method of coding was used to evaluate the caregivers’ behavior. The total IRS score was the total score of the child plus the total score of the caregiver.

## RESULTS

Table 1, 2 show the frequencies of items on the Interaction Rating Scale for 18-month-old, 30-month-old, 42-month-old and 7-year-old children.

**Table 1.** Frequencies of child items on Interaction Rating Scale (18M, 30M, 42M, 7Y)

Items	Categories	18M		30M		42M		7Y	
			
		*n*	%	*n*	%	*n*	%	*n*	%
	Total	231	100.0	344	100.0	207	100.0	82	100.0

**1. AUTONOMY**^a^ Child initiates interaction with caregiver.	not evident at all	0	0.0	1	0.3	0	0.0	0	0.0
	not evident	0	0.0	7	2.0	1	0.5	2	2.4
	neutral	20	8.7	24	7.0	14	6.8	2	2.4
	evident	80	34.6	118	34.3	108	52.1	29	35.4
	evident at high level	131	56.7	194	56.4	84	40.6	49	59.8

1. Child vocalizes while looking at the task materials.	No	24	10.4	4	1.2	1	0.5	0	0.0
	Yes	207	89.6	340	98.8	206	99.5	82	100.0
2. Child smiles or laughs during the episode.	No	70	30.3	21	6.1	5	2.4	7	8.5
	Yes	161	69.7	323	93.9	202	97.6	75	91.5
3. Child attempts to make eye contact with caregiver.	No	40	17.3	56	16.3	9	4.4	19	23.2
	Yes	191	82.7	288	83.7	198	95.7	63	76.8
4. Child initiates interaction with caregiver spontaneously.	No	6	2.6	2	0.6	0	0.0	0	0.0
	Yes	225	97.4	342	99.4	207	100.0	82	100.0
5. Child attempts to elicit caregiver’s response.	No	50	21.7	52	15.1	23	11.1	5	6.1
	Yes	181	78.4	292	84.9	184	88.9	77	93.9

**2. RESPONSIVENESS**^b^ Child is responsive to caregiver’s behavioral cues.	not evident at all	0	0.0	1	0.3	0	0.0	0	0.0
	not evident	3	1.3	6	1.7	6	2.9	4	4.9
	neutral	15	6.5	37	10.8	23	11.1	5	6.1
	evident	77	33.3	119	34.6	110	53.1	22	26.8
	evident at high level	136	58.9	181	52.6	68	32.9	51	62.2

1. Child displays strong reaction during the interaction	No	4	1.7	7	2.0	0	0.0	0	0.0
	Yes	227	98.3	337	98.0	207	100.0	82	100.0
2. Child gazes at caregiver’s face or task materials after caregiver’s non-verbal behaviors.	No	4	1.7	6	1.7	3	1.5	1	1.2
	Yes	227	98.3	338	98.3	204	98.5	81	98.8
3. Child looks at caregiver’s face or eyes when caregiver attempts eye contact.	No	55	23.8	107	31.1	20	9.7	42	51.2
	Yes	176	76.2	237	68.9	187	90.3	40	48.8
4. Child vocalizes or babbles within five seconds after caregiver’s verbalization.	No	44	19.1	3	0.9	2	1.0	0	0.0
	Yes	187	81.0	341	99.1	205	99.0	82	100.0
5. Child vocalizes or babbles within five seconds of caregiver’s gestures, touch, or changes in facial expression.	No	65	28.1	15	4.4	6	2.9	2	2.4
	Yes	166	71.9	329	95.6	201	97.1	80	97.6

**3. EMPATHY**^c^ Child behaves in accord with caregiver’s affective expression.	not evident at all	0	0.0	2	0.6	0	0.0	3	3.7
	not evident	3	1.3	25	7.3	15	7.3	2	2.4
	neutral	26	11.3	70	20.4	44	21.3	9	11.0
	evident	49	21.2	102	29.6	95	45.8	32	39.0
	evident at high level	153	66.2	145	42.1	53	25.6	36	43.9

1. Child gives, shows, or points to task material to share emotion with caregiver.	No	32	13.9	57	16.6	18	8.7	11	13.4
	Yes	199	86.2	287	83.4	189	91.3	71	86.6
2. Child looks at caregiver’s face to gather information/gain understanding.	No	63	27.3	128	37.2	30	14.5	34	41.5
	Yes	168	72.7	216	62.8	177	85.5	48	58.5
3. Child vocalizes or adjusts own behavior within five seconds in response to caregiver’s verbalization. (more than 50% of the time)	No	13	5.6	38	11.1	23	11.1	5	6.1
	Yes	218	94.4	306	89.0	184	88.9	77	93.9
4. Child smiles at caregiver within five seconds of caregiver’s verbalization.	No	105	45.5	50	14.5	5	2.4	17	20.7
	Yes	126	54.6	294	85.5	202	97.6	65	79.3
5. Child behaves within five seconds in accord with caregiver’s gestures, touch, or changes in expression.	No	52	22.5	72	20.9	59	28.5	4	4.9
	Yes	179	77.5	272	79.1	148	71.5	78	95.1

**4. MOTOR REGULATION**^d^ Child’s behavior is clearly directed toward the task and he/she is not overactive/underactive.	not evident at all	0	0.0	1	0.3	0	0.0	3	3.7
	not evident	6	2.6	6	1.7	9	4.4	2	2.4
	neutral	25	10.8	22	6.4	34	16.4	5	6.1
	evident	65	28.1	100	29.1	103	49.7	11	13.4
	evident at high level	135	58.5	215	62.5	61	29.5	61	74.4

1. Child widens eyes and/or shows postural attention to task situation.	No	1	0.4	3	0.9	0	0.0	0	0.0
	Yes	230	99.6	341	99.1	207	100.0	82	100.0
2. Child becomes appropriately active in response to task situation.	No	2	0.9	6	1.7	0	0.0	0	0.0
	Yes	229	99.1	338	98.3	207	100.0	82	100.0
3. Child’s movements are clearly directed toward/away from the task or task material.	No	1	0.4	1	0.3	0	0.0	1	1.2
	Yes	230	99.6	343	99.7	207	100.0	81	98.8
4. Child makes clearly recognizable hand motions towards task materials during the episode. (60% or more of the time)	No	2	0.9	7	2.0	4	1.9	2	2.4
	Yes	229	99.1	337	98.0	203	98.1	80	97.6
5. Child is neither restless nor overactive.	No	24	10.4	64	18.6	70	33.8	10	12.2
	Yes	207	89.6	280	81.4	137	66.2	72	87.8

**5. EMOTIONAL REGULATION**^e^ Child adjusts his/her emotional state to a comfortable level.	not evident at all	1	0.4	0	0.0	0	0.0	1	1.2
	not evident	12	5.2	16	4.7	12	5.8	2	2.4
	neutral	25	10.8	54	15.7	47	22.7	4	4.9
	evident	80	34.7	112	32.5	88	42.5	22	26.8
	evident at high level	113	48.9	162	47.1	60	29.0	53	64.7

1. Child stops displaying distress cues without caregiver’s response.	No	86	37.2	126	36.6	73	35.3	12	14.6
	Yes	145	62.8	218	63.4	134	64.7	70	85.4
2. Child stops displaying distress cues without caregiver’s soothing attempts.	No	70	30.3	87	25.3	45	21.7	4	4.9
	Yes	161	69.7	257	74.7	162	78.3	78	95.1
3. Child stops displaying distress cues within 15 seconds after caregiver’s soothing attempts.	No	1	0.4	49	14.2	28	13.5	3	3.7
	Yes	230	99.6	295	85.8	179	86.5	79	96.3
4. Child asks caregiver for help or consolation.	No	8	3.5	13	3.8	6	2.9	0	0.0
	Yes	223	96.5	331	96.2	201	97.1	82	100.0
5. Child is not startled by caregiver’s movements or changes in his/her facial expression.	No	3	1.3	0	0.0	0	0.0	1	1.2
	Yes	228	98.7	344	100.0	207	100.0	81	98.8

^a–e^: Tukey-Kramer multiple comparison tests (behavioral total score was used).

^a^18 < 30 < 42, 18 < 7Y, ^b^18 < 30 < 42, 7Y < 42, ^c^18 < 42, 30 < 42, ^d^42 < 30, 42 < 18, 42 < 7Y, ^e^18 < 7Y, 30 < 7Y, 42 < 7Y.

**Table 2.** Frequencies of caregiver items and overall evaluation on Interaction Rating Scale (18M, 30M, 42M, 7Y)

Items	Categories	18M		30M		42M		7Y	
			
		*n*	%	*n*	%	*n*	%	*n*	%
	Total	231	100.0	344	100.0	207	100.0	82	100.0

**6. RESPECT FOR AUTONOMY DEVELOPMENT**^f^ Partner encourages child’s autonomy.	not evident at all	0	0.0	0	0.0	0	0.0	0	0.0
	not evident	5	2.2	6	1.7	8	3.9	8	9.8
	neutral	9	3.9	37	10.8	29	14.0	16	19.5
	evident	49	21.2	138	40.1	110	53.1	27	32.9
	evident at high level	168	72.7	163	47.4	60	29.0	31	37.8

1. Caregiver allows child to explore task material for at least five seconds before providing first task related instruction.	No	25	10.8	3	0.9	7	3.4	0	0.0
	Yes	206	89.2	341	99.1	200	96.6	82	100.0
2. Caregiver pauses when child initiates behaviors during episode.	No	3	1.3	8	2.3	22	10.6	5	6.1
	Yes	228	98.7	336	97.7	185	89.4	77	93.9
3. Caregiver asks for no more than three repetitions when child is successful at completing the task.	No	5	2.2	5	1.5	8	3.9	0	0.0
	Yes	226	97.8	339	98.6	199	96.1	82	100.0
4. Caregiver does not physically force child to complete task.	No	21	9.1	20	5.8	20	9.7	13	15.9
	Yes	210	90.9	324	94.2	187	90.3	69	84.2
5. Caregiver halts the episode when child is distressed.	No	14	6.1	27	7.9	22	10.6	11	13.4
	Yes	217	93.9	317	92.2	185	89.4	71	86.6
6. After giving instructions, caregiver allows at least five seconds for child to attempt task before intervening.	No	16	6.9	11	3.2	17	8.2	23	28.1
	Yes	215	93.1	333	96.8	190	91.8	59	72.0
7. Caregiver allows non-task manipulation of task materials after the original presentation.	No	15	6.5	8	2.3	24	11.6	2	2.4
	Yes	216	93.5	336	97.7	183	88.4	80	97.6
8. Caregiver does not make critical or negative comments about child’s task performance.	No	11	4.8	13	3.8	12	5.8	6	7.3
	Yes	220	95.2	331	96.2	195	94.2	76	92.7
9. Caregiver encourages and/or allows child to perform task at least once before intervening.	No	4	1.7	1	0.3	3	1.5	9	11.0
	Yes	227	98.3	343	99.7	204	98.5	73	89.0

**7. RESPECT FOR RESPONSIVENESS DEVELOPMENT**^g^ Partner encourages child’s responsiveness.	not evident at all	0	0.0	0	0.0	0	0.0	1	1.2
	not evident	2	0.9	1	0.3	2	1.0	2	2.4
	neutral	5	2.2	27	7.9	25	12.1	13	15.9
	evident	56	24.2	133	38.6	123	59.4	25	30.5
	evident at high level	168	72.7	183	53.2	57	27.5	41	50.0

1. Caregiver positions child to safely support it.	No	1	0.4	11	3.2	21	10.1	0	0.0
	Yes	230	99.6	333	96.8	186	89.9	82	100.0
2. Caregiver provides an environment free of distractions.	No	3	1.3	3	0.9	5	2.4	2	2.4
	Yes	228	98.7	341	99.1	202	97.6	80	97.6
3. Caregiver positions child so it can reach and manipulate materials.	No	0	0.0	1	0.3	3	1.5	0	0.0
	Yes	231	100.0	343	99.7	204	98.5	82	100.0
4. Caregiver seeks the child’s attention before beginning the task, at the outset of the teaching interaction.	No	7	3.0	13	3.8	12	5.8	5	6.1
	Yes	224	97.0	331	96.2	195	94.2	77	93.9
5. Caregiver gives instruction only when the child is attentive (90% of the time).	No	9	3.9	69	20.1	40	19.3	16	19.5
	Yes	222	96.1	275	79.9	167	80.7	66	80.5
6. Caregiver positions child so eye contact is possible during the teaching period (60%).	No	12	5.2	0	0.0	2	1.0	1	1.2
	Yes	219	94.8	344	100.0	205	99.0	81	98.8
7. Caregiver changes position of child and/or materials after unsuccessful attempts by the child to do the task.	No	6	2.6	8	2.3	7	3.4	4	4.9
	Yes	225	97.4	336	97.7	200	96.6	78	95.1
8. Caregiver keeps child in visual range.	No	1	0.4	0	0.0	0	0.0	2	2.4
	Yes	230	99.6	344	100.0	207	100.0	80	97.6
9. Caregiver stays close to child and pays good attention.	No	0	0.0	0	0.0	2	1.0	6	7.3
	Yes	231	100.0	344	100.0	205	99.0	76	92.7

**8. RESPECT FOR EMPATHY DEVELOPMENT**^h^ Partner encourages child’s empathy development.	not evident at all	0	0.0	0	0.0	0	0.0	0	0.0
	not evident	3	1.3	2	0.6	5	2.4	8	9.8
	neutral	13	5.6	42	12.2	32	15.5	20	24.3
	evident	72	31.2	150	43.6	117	56.5	41	50.0
	evident at high level	143	61.9	150	43.6	53	25.6	13	15.9

1. Caregiver praises child’s efforts at least once during the episode.	No	65	28.1	86	25.0	50	24.2	49	59.8
	Yes	166	71.9	258	75.0	157	75.8	33	40.2
2. Caregiver emits positive, sympathetic, or soothing verbalizations.	No	11	4.8	11	3.2	3	1.5	9	11.0
	Yes	220	95.2	333	96.8	204	98.5	73	89.0
3. Caregiver smiles, or touches child within five seconds after child’s smile or vocalization (more than 90% of the time).	No	34	14.7	9	2.6	8	3.9	6	7.3
	Yes	197	85.3	335	97.4	199	96.1	76	92.7
4. Caregiver emits soothing non-verbal response (ie, pat, touch, rock, caress, kiss).	No	34	14.7	237	68.9	166	80.2	70	85.4
	Yes	197	85.3	107	31.1	41	19.8	12	14.6
5. Caregiver diverts the child by playing games, introducing new toy.	No	69	29.9	52	15.1	35	16.9	21	25.6
	Yes	162	70.1	292	84.9	172	83.1	61	74.4
6. Caregiver does not vocalize to the child while the child is vocalizing.	No	0	0.0	1	0.3	8	3.9	2	2.4
	Yes	231	100.0	343	99.7	199	96.1	80	97.6
7. Caregiver verbally praises child during the episode.	No	78	33.8	101	29.4	77	37.2	46	56.1
	Yes	153	66.2	243	70.6	130	62.8	36	43.9
8. Caregiver smiles and/or nods at the child.	No	32	13.9	4	1.2	3	1.5	5	6.1
	Yes	199	86.2	340	98.8	204	98.5	77	93.9
9. Caregiver responds to child’s vocalizations with affectionate verbal response.	No	20	8.7	16	4.7	21	10.1	6	7.3
	Yes	211	91.3	328	95.4	186	89.9	76	92.7

**9. RESPECT FOR COGNITIVE DEVELOPMENT**^i^ Caregiver encourages child’s cognitive development.	not evident at all	0	0.0	0	0.0	0	0.0	2	2.4
	not evident	14	6.1	11	3.2	7	3.4	9	11.0
	neutral	76	32.9	118	34.3	40	19.3	19	23.2
	evident	86	37.2	154	44.8	132	63.8	40	48.8
	evident at high level	55	23.8	61	17.7	28	13.5	12	14.6

1. Caregiver focuses own attention and child’s attention on task during most of the episode (at least 60% of the time).	No	18	7.8	26	7.6	4	1.9	7	8.5
	Yes	213	92.2	318	92.4	203	98.1	75	91.5
2. Caregiver describes perceptual qualities of task materials to child.	No	186	80.5	169	49.1	66	31.9	31	37.8
	Yes	45	19.5	175	50.9	141	68.1	51	62.2
3. Caregiver uses at least two different sentences or phrases to describe task to child.	No	128	55.4	195	56.7	53	25.6	22	26.8
	Yes	103	44.6	149	43.3	154	74.4	60	73.2
4. Caregiver uses explanatory verbal style more than imperative style in episode.	No	3	1.3	5	1.5	6	2.9	3	3.7
	Yes	228	98.7	339	98.6	201	97.1	79	96.3
5. Caregiver’s instructions are clear and unambiguous.	No	98	42.4	188	54.7	57	27.5	22	26.8
	Yes	133	57.6	156	45.4	150	72.5	60	73.2
6. Caregiver uses both verbal description and non-verbal instruction	No	6	2.6	12	3.5	14	6.8	1	1.2
	Yes	225	97.4	332	96.5	193	93.2	81	98.8
7. Caregiver uses teaching loops (alerting, instruction, performance, and feedback) in instructing child.	No	81	35.1	66	19.2	28	13.5	1	1.2
	Yes	150	64.9	278	80.8	179	86.5	81	98.8
8. Caregiver signals completion of task to child verbally or non-verbally.	No	122	52.8	113	32.9	59	28.5	43	52.4
	Yes	109	47.2	231	67.2	148	71.5	39	47.6
9. Length of caregiver instruction to child is age appropriate.	No	22	9.5	5	1.5	7	3.4	8	9.8
	Yes	209	90.5	339	98.6	200	96.6	74	90.2

**10. RESPECT FOR SOCIAL-EMOTIONAL DEVELOPMENT**^j^ Caregiver encourages child’s social-emotional development.	not evident at all	0	0.0	0	0.0	0	0.0	0	0.0
	not evident	2	0.9	7	2.1	7	3.4	6	7.3
	neutral	22	9.5	41	11.9	23	11.1	4	4.9
	evident	96	41.6	148	43.0	122	58.9	45	54.9
	evident at high level	111	48.0	148	43.0	55	26.6	27	32.9

1. Caregiver does not make negative comments to the child.	No	8	3.5	16	4.7	15	7.3	3	3.7
	Yes	223	96.5	328	95.4	192	92.7	79	96.3
2. Caregiver does not yell at the child.	No	1	0.4	1	0.3	1	0.5	0	0.0
	Yes	230	99.6	343	99.7	206	99.5	82	100.0
3. Caregiver does not use abrupt movements or rough handling.	No	2	0.9	5	1.5	1	0.5	1	1.2
	Yes	229	99.1	339	98.6	206	99.5	81	98.8
4. Caregiver does not slap, hit, or spank.	No	0	0.0	2	0.6	0	0.0	2	2.4
	Yes	231	100.0	342	99.4	207	100.0	80	97.6
5. Caregiver does not make negative comments to observer about the child.	No	9	3.9	0	0.0	2	1.0	2	2.4
	Yes	222	96.1	344	100.0	205	99.0	80	97.6
6. Caregiver’s body posture is relaxed during the episode (more than 50% of the time).	No	0	0.0	7	2.0	4	1.9	3	3.7
	Yes	231	100.0	337	98.0	203	98.1	79	96.3
7. Caregiver places him/herself in a face-to-face position with the child when talking to the child (more than 50% of the time).	No	40	17.3	0	0.0	0	0.0	1	1.2
	Yes	191	82.7	344	100.0	207	100.0	81	98.8
8. Caregiver behaves affectionately to child during the episode.	No	3	1.3	4	1.2	6	2.9	3	3.7
	Yes	228	98.7	340	98.8	201	97.1	79	96.3
9. Caregiver makes constructive or encouraging statements to the child during episode.	No	197	85.3	192	55.8	79	38.2	43	52.4
	Yes	34	14.7	152	44.2	128	61.8	39	47.6

* OVERALL IMPRESSION: A SYNCHRONOUS RELATIONSHIP^k^	not evident at all	0	0.0	0	0.0	0	0.0	1	1.2
	not evident	8	3.5	15	4.4	10	4.8	4	4.9
	neutral	36	15.6	72	20.9	44	21.3	20	24.4
	evident	102	44.1	148	43.0	107	51.7	42	51.2
	evident at high level	85	36.8	109	31.7	46	22.2	15	18.3

^f–k^: Tukey-Kramer multiple comparison tests (behavioral total score was used).

^f^7Y < 18 < 30, 42 < 30, ^g^42 < 18, 7Y < 18, ^h^7Y < 18, 7Y < 30, 7Y < 42, ^i^18 < 30 < 42, 18 < 7Y, 7Y < 42, ^j^18 < 30, 18 < 42, 18 < 7Y, ^k^18 < 30, 18 < 42, 7Y < 42.

Significant age differences were found on the subscales of autonomy, and emotional regulation. Autonomy at 30 months, 42 months, and 7 years was significantly higher than at 18 months. Seven-year-old children had significantly higher emotional regulation than the 18, 30, and 42-month-old children.

Other age differences were that older children used significantly more verbal responsiveness. There were also age differences among specific items, revealing important differences, for example, in types of interactions. Younger children and caregivers were more likely to demonstrate empathy through reference to a common thing (eg “look at the bird”), while older children were more able to respond to non-verbal cues, such as the nodding or eye movements of the caregiver.

## DISCUSSION

This study provides evidence that the IRS can be used as a reliable, valid, feasible and practical tool for the studies of caregiver-child interaction over time.^15^

First of all, the analysis of the IRS by age showed that IRS has high validity for cohort studies, because it can be used with the same subscales framework across ages from infants to 8-year-old.

Secondly, the IRS can be used in international comparative studies, because it is based on the most common frameworks used all over the world. The child subscales are based on various categories which are widely used in the research of social skills indicators. Also the caregiver’s subscales are based on the Home Observation for Measurement of the Environment (HOME), which has been widely used.

Third, we have evidence of the IRS in terms of discriminant validity for pervasive development disorder (PDD), attention-deficit/hyperactivity disorder (ADHD) and abused children. Children with PDD, ADHD, and abused children had lower levels of empathy and self-control in areas such as motor regulation and emotional regulation compared to children without these conditions.^13^

Fourth, the IRS has high correlations with the SDQ (Strength and Difficulties Questionnaire), and high reliability.^16^ There were significant correlations between the “empathy”, “motor regulation”, “emotional regulation”, caregiver’s “Respect for responsiveness” in the IRS and the “hyperactivity-inattention domain” in the SDQ. Also, “autonomy”, “responsiveness”, “empathy” in the IRS and less “peer problems domain” in the SDQ, “responsiveness”, “empathy”, “motor regulation” in the IRS and “prosocial behavior domain” in the SDQ, caregiver’s “respect for empathy development” in the IRS and less total difficulties scores in the SDQ.

While the IRS provides valuable insights, it is also important to acknowledge its limitations. First, the IRS subscales might not cover all dimensions of social skills, although we used the most common frameworks of social skills. Second, while the IRS expects to using same scoring standard from birth to eight years old as a standardized tool in cohort studies, different developmental features of items across developmental stages might be better to take into consideration. Despite these limitations, the IRS can be considered an established, valid screening instrument reflecting child-related attributes of the caregiver-child interaction. It provides evidences of the fact that in order to study children’s social development, it is important to evaluate various features of the caregiver-child interaction as a measure of social skills.

We are in the process of analyzing data of 42-month-old. Further research has the potential to reveal the features of the caregiver-child interaction development, and enhance knowledge of implications for caregivers and child-care professionals.

## APPENDIX

### Japan Children’s Study Group

Chairman: Zentaro Yamagata (Department of Health Sciences, School of Medicine, University of Yamanashi), Hideaki Koizumi (Advanced Research Laboratory, Hitachi, Ltd.).

Participating Researchers: Kevin K. F. Wong, Yoko Anji, Hiraku Ishida, Mizue Iwasaki, Aya Kutsuki, Misa Kuroki, Haruka Koike, Daisuke N Saito, Akiko Sawada, Yuka Shiotani, Daisuke Tanaka, Shunyue Cheng, Hiroshi Toyoda, Kumiko Namba, Tamami Fukushi, Tomoyo Morita, Hisakazu Yanaka (Research Institute of Science and Technology for Society, Japan Science and Technology Agency), Yoichi Sakakihara (Department of Child Care and Education, Ochanomizu University), Kanehisa Morimoto (Graduate School of Medicine, Osaka University), Kayako Nakagawa (Graduate School of Engineering, Osaka University), Shoji Itakura (Graduate School of Letters, Kyoto University), Kiyotaka Tomiwa (Graduate School of Medicine, Kyoto University), Shunya Sogon (The Graduate Divisiton of the faculty of Human Relations, Kyoto Koka Women’s University), Toyojiro Matsuishi (Department of Pediatrics and Child Health, Kurume University), Tamiko Ogura (Graduate School of Humanities, Kobe University), Masako Okada (Koka City Educational Research Center), Hiroko Ikeda (National Epilepsy Center Shizuoka Institute of Epilepsy and Neurological Disorder), Norihiro Sadato (National Institute for Physiological Sciences, National Institutes of Natural Sciences), Mariko Y. Momoi, Hirosato Shiokawa, Takanori Yamagata (Department of Pediatrics., Jichi Medical University), Tadahiko Maeda, Tohru Ozaki (The Institute of Statistical Mathematics, Research Organization of Information and Systems), Tokie Anme (Graduate School of Comprehensive Human Sciences, University of Tsukuba), Takahiro Hoshino (Graduate School of Arts and Sciences, The University of Tokyo), Osamu Sakura (Interfaculty Initiative in Information Studies, The University of Tokyo), Yukuo Konishi (Department of Infants’ Brain & Cognitive Development, Tokyo Women’s Medical University), Katsutoshi Kobayashi (Center for Education and Society, Tottori University), Tatsuya Koeda, Toshitaka Tamaru, Shinako Terakawa, Ayumi Seki, Ariko Takeuchi (Faculty of Regional Sciences, Tottori University), Hideo Kawaguchi (Advanced Research Laboratory, Hitachi, Ltd.), Sonoko Egami (Hokkaido University of Education), Yoshihiro Komada (Department of Pediatric and Developmental Science, Mie University Graduate School of Medicine Institute of Molecular and Experimental Medicine), Hatsumi Yamamoto, Motoki Bonno, Noriko Yamakawa (Clinical Research Institute, Mie-chuo Medical Center, National Hospital Organization), Masatoshi Kawai (Institute for Education, Mukogawa Women’s University), Yuko Yato (College of Letters, Ritsumeikan University), Koichi Negayama (Graduate School of Human Sciences, Waseda University).
